# Treating and Capping Exposed Nerves

**Published:** 1860-12

**Authors:** W. A. Pease


### TREATING AND CAPPING EXPOSED NERVES.

BY DR. W. A. PEASE.

Dr. Watt has taken the initiative, in bringing before the readers of the profession, a subject that excited a lively interest and discussion at Saratoga, viz: the propriety of treating and capping the exposed nerves of teeth. As a whole, I am pleased with his article, and, though it is apparent he has a kind feeling for the nerves, and evidently wishes them a long life and no aches, and, indeed, is willing to give them a helping hand, and by trusting them, give them a chance for their lives, it is obvious he does so with some mental misgiving and many fears. As this subject will command the attention of the profession for some time to come, it will be proper for observers to put on record the results of their experiments and experience, and such other observations as may be pertinent, or will tend to throw light upon the subject.

All admit that the nerve (using a comprehensive term) is capable of depositing dentine, or toothbone. From childhood up to a certain age, the nerve recedes from the crown, or cutting edge of the tooth, and deposits dentine as it goes, till it arrives at a certain point, which is generally half way between the cutting edge of the tooth and the gum, where it generally remains stationary during life, unless, from some irritating cause, it is again aroused to action, again to deposit dentine, or calcific matter. The deposition of dentine seems to be solely due to the periosteum, or the membrane lining the nerve canal; but, at times, there is another and inferior material deposited, without much reference to use, and for no intelligible purpose. This inferior material, or calcific matter, is deposited capriciously and at random, often near the foramen, or scattered along, at different points, in the body of the nerve, in what may be called nodules, or points of diffusion. Where the circumstances are favorable, these nodules increase, the deposit following along the course of the fiber, or vessel on which the nodule is situated, till, finally, the fibers are all calcified, when they are united together, forming one calcified mass. Then the periosteum may become ossified and unite the calcified nerve to the tooth. It is needless to say that these deposits are exceedingly rare, and when they do occur, they must probably be referred to those morbid, or diathetic conditions of the system, in which we find valvular, or muscular ossification. Certain it is that this calcification is of no practical value to the dentist; but he possibly may yet derive some benefit from the deposition of dentine by the periosteum. The one seems to be systemic, or diathetic, but little due to caries or dental irritation, and may be also going on in the arteries or tissues outside of the teeth; the other is chiefly due to local and dental irritation, and is the nearest approximation we meet in the teeth of that osseous repair which we know follows injury of the bones in other parts of the system. Reasoning from analogy, from the known laws that govern the repair of injury of the long bones, we may expect too much, and perform operations that will end disastrously, when a more intimate knowledge of dental pathology would save us from mortification. It is well known that the periosteum is a secretory organ; it forms the bones, and, to a certain extent, repairs their injuries; it unites most fractures, or resections; but there is no authentic case where the fangs of a tooth, after fracture, have been united. So necessary is it to the health of bone, that a part denuded of it is liable to necrosis; and the most that can be said of it is, as a dental organ, that in some constitutions and under certain circumstances, it deposits dentine to prevent its exposure by the gradual loss of the substance of the tooth, from external attrition or caries; and, in some few instances, it is reported to have closed an opening, made by a dentist, in preparing a cavity, and to have deposited a septum of bone between itself and a plug. Thus, so far as we know, its reparatory capabilities are exceedingly limited, and seem to be exceptional rather than constant; and, it is probable, they will never be of much benefit to man, except in those cases, where, from direct antagonism, the teeth are gradually worn away during a series of years. In such cases, there is generally a secondary deposit of dentine of undoubted value.

Here the question arises, can the dentist call into exercise, at will, the secretory properties of the periosteum, known to be active during the gradual wearing away of the teeth; for it is known that some stimulus is necessary, as the nerve rarely makes any effort to protect itself from the ravages of caries, and the few cases where this effort is made, simulate the gradual wasting of the teeth from mastication. If we go to analogy for an answer, we find the requisites to unite a fracture are apposition and rest; of resection, the preservation of the periosteum; and recent experiments have shown that the periosteum, transplanted to other structures, as the comb of a cock, still performs its legitimate function, and deposits ossific matter. When there is an indolence of the secretory function, and a failure to unite a fracture, or rather, when the callus has been broken, or the granulation interrupted, the surgeon, after cutting down to the bone, removes a portion of the ends, or drills holes into them, and places therein pegs of ivory or bone, to re-excite an inflammation, after which, the fracture generally unites. Here the ivory pegs perform the same office that the grain of sand does in the shell of the oyster; and it is possible that some foreign substance, placed against the internal periosteum of a tooth, under favorable circumstances, may excite it to action. This is the more probable, inasmuch as some stimulus is known to be required, as caries seldom excites it to deposit osteo-dentine. Those who have split many carious teeth will not require to be told that periosteal depositions are exceedingly rare; and that class of dentists who destroy the nerve and remove it from the tooth, will bear witness to the infrequency of periosteal deposits. For myself, I can say I have removed the nerve from its canal, almost daily for many years, and as it comes from the canal and hangs dangling from the point of the instrument, I have examined it, called the attention of my patient to it, (for it was satisfactory to him to know that the nerve was not only dead, but actually removed from the tooth), and together we have examined it, held it up between us and the light, and remarked how it represented the exact form of the nerve canal. We have placed it on paper, and there re-examined and handled it, before it was taken away by the patient as a curiosity; and I must confess I have never met with a single case of calcification, or ossific deposit by the nerve, attributable to caries. Cases there may have been, where, at some point beneath the cavity, the nerve has made a feeble effort to protect itself. If so, they have escaped my observation; and I think I should have noticed them, as in excavating a cavity, when I approach that point where anatomy says the nerve should be, I have almost held my breath, lest an incautious cut should expose it; and if, from accident or necessity, that point was reached, I have invariably found the nerve, or the fetid chamber where it died. From this, I am persuaded, as well as from all the information I can derive from others, if I except some few cases to be mentioned in another place, that the efforts of the nerve to protect itself from caries are so slight and so rare, in comparison to the numberless cases met with every year where no effort has been made, that they scarcely arise to the dignity of an exception.

The question then arises, if caries is removed by thorough excavation, the nerve is exposed and healthy, have we any known means to stimulate that nerve to take care of itself, by depositing a septum of bone between itself and the plug? Is the plug of itself a sufficient stimulant, as is the grain of sand in the shell of the oyster; or must we resort to other means ? If so, what? Medication ? What are the agents, or is rest sufficient? Hitherto it has been supposed that the nerve was simply exposed, but not wounded. If wounded, would that complicate the case? If so, how much? What would be the prognosis; or would that depend upon the nature of the wound, whether it was a point, or a line, a simple slit, leaving the edges of the periosteum in apposition, or whether there was an actual loss of substance? If there is merely a slit, will it heal kindly by first intention; and will that be just enough inflammation to ensure the necessary deposit? Or, if there is a loss of substance, will that place heal and be restored? If so, how? By granulation? Then there must be secretion, effusion. Where is that secretion to go, after the tooth is thoroughly plugged; and what is to become of it, if it finds lodgement? Will it putrefy, or be absorbed? Here a delicate question arises. How much irritation or inflammation can we set up in the nerve, encased as it is in a solid wall of bone, and not produce engorgement, or gangrene? And provided there should be too much phlogiston, have we any special anti-phlogistics to reduce it?

I have before remarked that I had seen cases where the nerve had deposited dentine of repair, to protect itself from caries. A few words will dispose of them, and show that they do not constitute an independent class, but really belong to that class in which the nerve deposits dentine of repair, to protect itself from the gradual wear of teeth in mastication. They were, so far as I recollect, cases where, at some time, caries had been active, but from some cause, generally from the removal of a tooth, where the cavity was on the approximal surface, the smaller cavity in the remaining tooth ceased to increase, and the decay lay dormant for several years. In plugging such teeth, I have often found secondary dentine, and in some of them also calcific deposits. Now, the long time in which caries has lain dormant in these teeth fulfills all the conditions that exist in the gradual wear of the teeth during mastication. It gives the nerve time to protect itself and shows that the only thing necessary, so far as we now know, to enable it to protect itself, by a secondary deposit of dentine, is time, or the gradual loss of substance of the tooth during several years. The past summer I was plugging some teeth for a gentleman of seventy years of age. While preparing the front approximal cavity of the second superior bicuspid tooth, I noticed he flinched. Supposing the nerve was dead, I had cut down boldly into the cavity to secure a good base, as the tooth was very badly decayed, and also to prepare the fang for filling. I examined it, and found a very healthy deposit of secondary dentine. Afterwards, while preparing a cavity for him in an incisor tooth, I was very cautious in my explorations, till I found the nerve was dead.

Without describing other cases, or discussing this question further at present, I will say, I have tried every plan my ingenuity could devise, or that has been suggested by others, and I do not know that I have plugged over a single exposed nerve that is now alive. Bear in mind, I do not say none of them are. Some of them have given no trouble; others, I know, are dead. Whether alive or dead, they only prove that the nerve, or teeth, will sometimes tolerate a great deal.

